# The Gendered, Misogynoiristic, and Colonial Genocidal Logics of Strip Searching

**DOI:** 10.1177/08861099241297153

**Published:** 2024-11-14

**Authors:** Jessica Hutchison

**Affiliations:** 1 Faculty of Social Work, Wilfrid Laurier University, Kitchener, ON, Canada

**Keywords:** Strip searching, abolition feminism, settler colonialism, misogynoir

## Abstract

Women with lived experience of strip searching have been calling for it to be banned as a practice for decades; however, it remains a routine practice in carceral settings such as prisons and jails. Given the mass incarceration of Indigenous women and disproportionate rate of Black women in federal prisons in Canada, an anti-racist and gendered anticolonial analysis of strip searching is warranted. Thus, this paper shares findings from conversations with 23 previously incarcerated women, the majority of whom are Black and Indigenous, about their experiences of being strip searched in prisons. The main theme throughout the conversations was that strip searching is sexual violence by the state. Furthermore, the harms of strip searching are gendered in that women are forced to remove their tampons and pads during menstruation to show guards. The paper also elucidates the ways in which strip searching enacts misogynoiristic and colonial genocidal logics historically rooted in projects such as Indian Residential Schools and the enslavement of Black women. It ends with a call for abolition feminist social work praxis by meeting the direct needs of women who are strip searched while also advocating for it to be banned as a practice.

Despite decades of critique and calls to abolish its use, strip searching remains a routine practice in carceral spaces such as jails, prisons, and police cells (among other sites) (Davis, 2003; George, 1993; Kilroy, 2003; McCulloch & George, 2009; Pereira, 2001). Women with lived experience of strip searching have been drawing attention to the gendered harms of strip searching and naming it what it is – sexual violence by the state (Davis, 2003; Kilroy, 2003; Shakur, 1987).

Given the hyper-incarceration of Indigenous women and disproportionate rate of Black women incarcerated in federal prisons in Canada (Office of the Correctional Investigator [OCI], 2022), an anti-racist and anti-colonial analysis is warranted. As such, this article reports on some of the gendered harms of strip searching and offers an expanded analysis into the misogynoiristic (Bailey, 2021) and colonial genocidal logics inherent in the strip searching of women in prison. Understanding the specific and nuanced harms of strip searching on Indigenous and Black women is essential for social workers engaging in decolonizing and anti-racist praxis. Thus, this research centers the voices of Indigenous and Black women and situates their experiences within the sociopolitical context of white supremacy and settler colonialism. Furthermore, it deepens the activist and scholarly critiques of strip searching as a gendered process and ends with a call to abolish it as a practice. It begins with an overview of what strip searching is, its stated purpose, and data on how much contraband^1^ is discovered through strip searching.

## Literature Review

### What is a Strip Search?

According to federal prison legislation and policy in Canada, a strip search is a “visual inspection of the naked body” (Corrections and Conditional Release Act [CCRA], 1992, s. 46) and can include requiring a woman to “open her mouth, display the soles of her feet, run her fingers through her hair, present open hands and arms, bend over or otherwise enable the staff member to perform the visual inspection” (CSC, 2017). The stated purpose of strip searching is, “to prevent the introduction and possession of contraband and unauthorized items” (Government of Canada, 2015). Two women guards participate in the strip searching process with one giving the commands and the other witnessing (CCRA, 1992, s.48). Legislation and policies differ across jurisdictions; however, the core process of guards looking at/in women's naked bodies is the same.

#### Strip Searching Rarely Discovers Items

**Figure 1.**
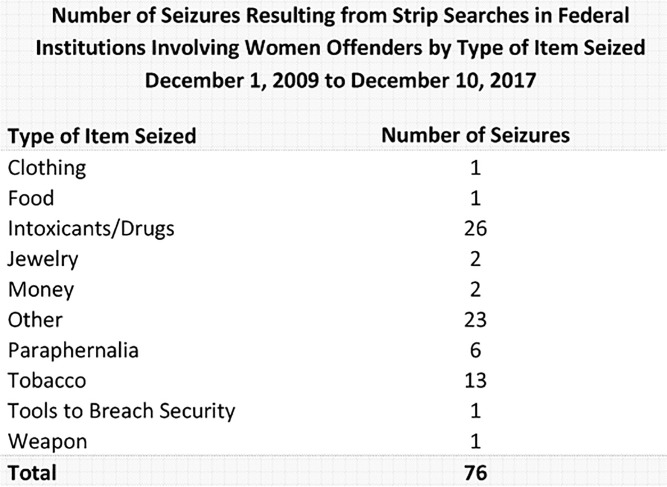
Strip Searching Data from the Correctional Service of Canada.

Data from prison authorities in Canada and Australia demonstrate miniscule numbers of items are discovered through strip searching women in prison. In 2017, the Canadian Association of Elizabeth Fry Societies (CAEFS) submitted an Access to Information and Privacy (ATIP) request to CSC asking for data on the number of strip searches performed in prisons designated for women^2^ since 2007 as well as the number and nature of any items found during a strip search. As can be seen in Figure 1 below, over an eight-year period, an average of less than two items per prison per year were found through strip searching in prisons designated for women (76 items across 6 federal prison sites over 8 years) (CSC, 2020).

These data from Canada are consistent with data from Australia, which have also been obtained through the ATIP process. For example, in an Australian prison for women with a daily average of 200 prisoners, over a one-year period, 18,889 strip searches were performed and only one item of contraband was found (McCulloch & George, 2009). Furthermore, in a prison with a daily average of 100 women, 13,000 strip searches were performed over a 1-year period and only 2 cigarettes were found in 2 randomly selected months.

A counterargument is that strip searching prevents items from entering the prison. Indeed, this was the claim by prison officials at Grand Valley Institution for Women (GVI), the largest federal prison designated for women in Canada, when other advocates for CAEFS and I challenged its use. However, despite GVI's policy to strip search everyone 100 percent of the time upon return to the prison from community in 2018, items were still entering the prison, which suggests that strip searching is not preventative.

### Abolition Feminist Analysis of Strip Searching

For over three decades, women with lived experience of imprisonment and strip searching have referred to strip searching as sexual assault and called for it to be banned as a practice (Davis, 2003; Kilroy, 2003; Shakur, 1987). Foundational to this international movement was the 2001 “Stop State Sexual Assault” campaign developed by Sisters Inside, an Australian feminist abolition organization that centers women with lived experience of incarceration (Davis et al., 2022; Kilroy, 2003). Sisters Inside's analysis of strip searching drew clear connections between state violence and intimate partner violence (Davis et al., 2022; Kilroy, 2003).

This analysis is central to the abolition feminist framework and praxis, which elucidates the connections between interpersonal and state violence and situates contemporary manifestations of violence within the historical roots of systems of domination and oppression (Davis et al., 2022; Kaba & Ritchie, 2022; Richie, 1996, 2012; Ritchie, 2017). Specifically, gender and sexual violence is situated within the histories of white supremacist, settler colonial nations such as Canada, the United States, and Australia.

#### Settler Colonial and Misogynoiristic Sexual Violence

As a settler colonial nation state, Canada was created and is maintained on the subordination and dispossession of Indigenous and Black bodies as settler colonial genocide, slavery, white supremacy, and patriarchy are intricately connected and mutually reinforcing. There is a long history of settler colonial states, including Canada, using sexual violence against Black and Indigenous women in pursuit of land and capital (Davis, 1981; Hartman, 1997; MMIWG Inquiry, 2019). In the settler nation state, Indigenous and Black women are rendered disposable (Davis, 1981; Razack, 2015; Reece, 2020) albeit for different reasons, but towards the same settler goal. As Black feminist Rai Reece (2020) writes, “Black and Indigenous peoples’ histories are often siloed, but it is important to make visible the specificities and uniqueness of anti-Black and anti-Indigenous racism as connected to land stolen from people and people stolen from land” (p. 1).

**Settler Colonial Sexual Violence.** Over 50 percent of people in prisons designated for women are Indigenous (OCI, 2022) despite making up less than five percent of the overall population in Canada (Government of Canada, 2024). In this way, prisons are a site through which Canada continues to enact colonial genocide against Indigenous peoples as concluded by the MMIWG Inquiry (2019). This conclusion is consistent with others’ claims that settler nations were founded on colonial genocidal logics with the goal of eradicating Indigenous peoples to steal and occupy their lands and resources (Anderson, 2016; Maynard & Simpson, 2022; Monchalin, 2016; Simpson, 2017).

The genocidal use of colonial sexual violence against Indigenous women has occurred since contact (Deer, 2015). The imposition of patriarchal subordination upon first contact provided the foundation through which Indigenous women's bodies were considered ‘rapeable’ as was, by extension, their land, which carries feminine qualities (i.e., Mother earth) (Deer, 2015; Deer & Warner, 2019; Monchalin, 2016). This gendered colonial sexual violence occurs in multiple forms across various sites, including in carceral settings such as prisons and jails, where Indigenous women are hyper-incarcerated.

**Misogynoiristic Sexual Violence.** Black women are uniquely subjected to a form of violence rooted in the intersection of gender and racial marginalization (Bailey, 2021). As such, Dr. Moya Bailey coined the term “misogynoir” which refers to “the anti-Black racist misogyny that Black women experience” (Bailey & Trudy, 2018, p. 762). Thus, Black women experience “misogynoiristic” violence as a result of misogynoir (Bailey, 2021, p. 2).

Black women are disproportionately incarcerated in prisons designated for women in Canada (OCI, 2022), and have been historically marginalized and excluded from analyses of state violence (Crenshaw, 2012; Maynard, 2017; Reece, 2007, 2010; Ritchie, 2017). However, Black women have been targets of the state for centuries in Canada. Despite attempts by the nation state to deny slavery existed in Canada or to minimize the horrendous conditions by comparing it to the United States, slavery was an institution in Canada for over 200 years, from 1628-1833 (Cooper, 2006; Reece, 2010). Further, Black women are marginalized in narratives about slavery in Canada despite enduring horrific misogynoiristic violence (Cooper, 2006). Misogynoiristic sexual violence has been used against Black women since the beginning of the transatlantic slave trade, with the institutionalization of rape acting as a foundation of slavery. While the degree and severity of slavery-driven rape of Black women and strip searching in modern prisons differs unquestionably, the parallels between Black women not having full autonomy and control over their bodies during slavery and in modern-day prisons is striking.

While there is a rich body of critical feminist social work research into various aspects of imprisonment and criminalization such as Kim (2018), Richie (1996, 2012) and Pollack (2007, 2009, 2012), there has yet to be attention to specific forms of state-inflicted sexual violence in prisons designated for women, particularly from an abolition feminist social work lens. However, there is an emerging scholarship exploring these issues, including a special issue of *Affilia: Feminist Inquiry in Social Work* dedicated to abolition/anti-carceral feminist social work frameworks and analyses.

Therefore, given the dearth of empirical research into strip searching in prisons designated for women, particularly in social work; the paucity of misogynoiristic and anti-colonial analyses of the use of strip searching; the mass incarceration of Indigenous women and disproportionate rate of Black women in prisons designated for women in Canada (OCI, 2022); and the marginalization of Black and Indigenous women in analyses of state violence (Crenshaw, 2012; Maynard, 2017; Monture-Angus, 1995; Reece, 2010; Ritchie, 2017), the following questions guided the research:

1. In what ways is strip searching women in prison a form of state-inflicted sexual violence?
2. How are gendered, misogynoiristic, and colonial genocidal logics embedded within the practice of strip searching women in prison?
3. What strategies do women engage in to resist the sexual violence enacted on them by the state?

The focus of this article is on question two: How are gendered, misogynoiristic, and colonial genocidal logics embedded within the practice of strip searching women in prison?

## Methods

### Positioning Myself

I have a responsibility to locate myself so readers can learn who I am, where I come from, and where I am going and thus can assess my trustworthiness regarding the pages ahead (Absolon, 2022; Jackson et al., 2024; Kovach, 2021; Wilson, 2008). This is part of my commitment to engaging in research grounded in relational accountability. I am a non-Indigenous, white, cisgender woman settler who has never been criminalized or imprisoned. My ancestors come from England, Ireland, and Scotland, and I am recently coming into this understanding of my family lineage, and why we came to settle on Turtle Island.

I recognize my positionality influences all aspects of my research including my epistemology, the topics I choose to explore, the authors I read (and do not read), the lens through which I interpret the literature and data, and what I deem relevant to my research. I also recognize my declaration of whiteness (Ahmed, 2004; Braun & Clarke, 2006), and other privileged social positions, does not absolve me from responsibility of benefitting from white supremacy, settler colonialism, and other structures of oppression. Rather, my scholarly work is an attempt to challenge, within myself and within the prison system, gendered, racist, and colonial logics. With the understanding that positionality is fluid and changes with sociopolitical contexts (Jacksonet al., 2024), throughout this research process I engaged in active reflexivity and decolonization to unpack the ways I may be complicit in the continuation of these types of practices.

It is also important to name how I came to this research topic (Wilson, 2008). I used to an advocate with CAEFS, which is a national, abolition feminist organization dedicated to advocacy for women, trans, non-binary, and Two-Spirit people who are criminalized and imprisoned. My role was to work directly with people who are incarcerated at GVI whereby I received phone calls from them, on an almost daily basis, usually requesting support for addressing various types of violence perpetrated against them by the state. My job was to determine how to most effectively address this violence, which included engaging with lawyers, advocating to the warden, and sometimes, just bearing witness to the countless types of violence women and gender-diverse people experienced daily. Over time, I became increasingly concerned about the gendered and racialized violence perpetrated by the state, often through legitimated and mandated policies and practices. It was one of the most inspiring, frustrating, soul-sucking, and soul-giving work experiences I have ever engaged in. It fundamentally shaped my choice to research this topic as strip searching was often identified by those imprisoned at GVI as a harmful practice they wanted help with abolishing. Similarly, it affirmed to me that relationships are central to knowledge production and that people with lived experience are valid knowers.

I intentionally developed this study from an anti-racist and decolonizing perspective in order to minimize the potential harm my research could cause participants (Smith, 1999; Tuck & Yang, 2014). Therefore, I sought out research methodologies that are epistemologically, theoretically, and axiologically consistent with this aim.^3^

### How I Engaged Participants

This study engaged a qualitative research design, which centered the voices and experiences of Indigenous and Black women, through virtual sharing talking and individual conversations. Given my research was grounded in relational accountability, I engaged participants through existing relationships, which helps to build rapport and places the researcher in a circle of relations with the participants (Wilson, 2008). I invited women I personally knew through my various roles in advocacy over the years, such as with CAEFS and a restorative justice agency called Community Justice Initiatives, and asked intermediaries to invite women with whom they are in relationship. Through doing this, the intermediaries were ‘vouching’ for me and indicating they trust me to engage in ethical and relational processes, and to act with integrity (Wilson, 2008). This differs from participant-driven recruitment in that it was not based on participants referring others to the study; rather, it was based on relationships I had with women as well as relationships others (who had not been previously incarcerated) had with women. This relational approach to recruitment is a common practice in Indigenous methodologies (Absolon, 2011, 2022; Wilson, 2008). This method has the potential to miss some diversity in experiences given there are many previously incarcerated women I and my intermediaries do not know; thus, their experiences were not captured in this study.

**Who I spoke with**. I spoke with 23 cisgender women who had been incarcerated in a Canadian federal prison but were now living in the community. Eleven identified as Indigenous,^4^ six as Black, five as white, and one as racialized^5^. Due to the global Covid-19 pandemic, talking conversation circles were held virtually using the platform Zoom. The virtual nature of the conversations provided the opportunity to expand the focus of my study throughout Canada and resulted in women participating from British Columbia (n = 3), Saskatchewan (n = 3), and Ontario (n = 17).

**How I gathered stories.** Given the mass incarceration of Indigenous women and disproportionate rate of Black women in prison (Maynard, 2017; OCI, 2022) I prioritized their experiences of strip searching in this study. Further, given the sensitivity of the topic I felt it was important to create as trusting and comfortable an environment as possible to promote authentic dialogue, solidarity, and respect (Absolon, 2011; Wilson, 2008). Black and Indigenous women have similar, yet unique and specific histories and experiences of interpersonal and state violence. Thus, providing a space for women to share these experiences without fear of being judged, and as a form of collective healing, was essential to my process of relational accountability. Accordingly, I hosted three conversation circles with Indigenous women, two circles with Black women, and one circle with white women.

I hosted six virtual small talking circles following Anishinaabe circle protocols as taught to me by Dr. Kathy Absolon,^6^ Anishinaabe Knowledge Keeper and Professor of Indigenous Social Work, with one round of open discussions. Talking circles provide a culturally congruent process for searching for knowledge in that they provide the opportunity for active engagement, and prevent people from interrupting, dominating, and otherwise monopolizing the conversation (Absolon, 2011). To facilitate a talking circle, people sit in a circle, and an open-ended question or topic is posed to the group to promote a conversational style format rather than a rigid and controlled atmosphere (Absolon, 2011). For a variety of reasons, five women were unable to participate in the talking circles. Thus, I spoke with four women in virtual one-on-one conversations via Zoom and one woman on the phone due to parole restrictions preventing her from using the internet.

**How I made meaning and fostered trustworthiness.** As Absolon (2011) explains, the methodological journey, including meaning making, is an organic process, which can emerge naturally throughout the course of the research. My meaning making phase engaged this organic process, resulting in a cumulative meaning making framework (Wilson, 2008) comprised of seven distinct but non-linear overlapping phases: 1) during the circle and individual conversations; 2) listening to the recorded conversations and reading the transcripts; 3) thematic analysis using qualitative analysis software NVivo; 4) listening to the audio recordings of the conversations while sitting outside a prison; 5) informally through conversations, reflection, and preparation for testifying as an expert witness on strip searching; 6) sharing findings chapters with participants for their feedback and input; and 7) cumulative meaning making.

Furthermore, I understand meaning making and trustworthiness of the meaning made to be intricately intertwined (Absolon, 2011; Wilson, 2008). Throughout the meaning making processes, I engaged in several emergent strategies for fostering the trustworthiness of how I was making meaning of the stories shared with me. Therefore, in the following section, I present the ways in which I engaged in meaning making and fostered trustworthiness. A more fulsome and detailed description of my methodology is provided in a methodological manuscript (Hutchison, 2024).

Cree scholar Shawn Wilson (2008) describes meaning making as occurring while story gathering is taking place. Thus, in phases one and two, I took handwritten notes of things women said that stood out to me, helped me think of things differently, or confirmed what I already thought/knew. In this way, I was allowing for an organic meaning making process (Absolon, 2011) that was grounded in my research questions and theoretical framework.

In phase three, I utilized thematic analysis (Braun & Clarke, 2006), which is a common meaning making process in many forms of qualitative research, including Indigenous and feminist research (Kovach, 2021; Wilson, 2008). I applied iterative analysis (Braun and Clarke, 2006) by applying my theoretical framework to what I was reading (deductive meaning making), as well as looking for other patterns and themes that were not part of my theoretical framework but were helpful in answering my research questions (inductive meaning making).

However, after completing the first three phases, I had a nagging feeling that something was missing. Anishinaabe scholar Kathy Absolon suggested I step away from the computer and go to a place where deep meaning could be made. I knew exactly where I needed to be for this to occur. I felt a visceral need to go to GVI where I had spent hundreds of hours inside advocating for people imprisoned there in my role with CAEFS. I literally felt as though I was being pulled by some force to the prison. I needed to *see* the prison; to *feel* the prison while engaging in deep listening to women's stories. Thus, this phase comprised of me driving to the prison, parking in the lot beside the prison as I knew prison authorities would never allow me access to their grounds, and walking up and down the long driveway that shared a fence with GVI while listening to the recorded conversations. While I was listening and walking, I took notes on my phone that struck me as important, those I had not heard before or heard differently during this session, and reminders to myself about things to follow up on. By not being tethered to my computer and beholden to the written word, listening to women's voices while in the presence of the violent prison apparatus enabled ways of knowing that would otherwise remain locked behind the colonial wall of knowledge. *Seeing* the prison and *feeling* the prison while *hearing* women speak about the abuses they experienced at the hands of the state provided a more accurate portrayal and understanding of the violence of strip searching.

Throughout the entire research process, I carried the stories women gifted me in my heart, mind, spirit, and body (Absolon, 2011; Wilson, 2008) and engaged in several different types of ‘informal’ meaning making to foster rigor and trustworthiness (Lincoln & Guba, 1985). Phase five comprised of peer-debriefing conversations with trusted colleagued and mentors, testifying at a trial about strip searching in a provincial jail, and constantly reflecting on the stories shared with me.

I engaged in member checking in phase six by asking each participant if they would like to read their quotes along with the context I wrote around them as I wanted to ensure I was presenting their words in the way they had intended (i.e., rigor and trustworthiness). It was during this phase I also asked each person if they would like to use their real first name or a pseudonym of their choosing. Approximately half of women I spoke with chose to use their real name; the others selected a fake name. Of the 18 women I was able to be in contact with, 17 asked to review their quotes. Only one made a small change to one of her quotes.

Finally, Wilson (2008) frames meaning making as ‘cumulative analysis,’ which is not a linear process, and is how my meaning making process organically occurred (Absolon, 2011). My meaning making process was not linear. While I had an initial idea of how I thought the meaning making would unfold, along the way, it began to take on different and unexpected shapes (i.e., prison listening and testifying in court). All of the pieces have come together in a way I believe they were meant to and in a way that stays true to the spirit and heart of women's sharing.

## Findings and Analysis

As I spoke with women, it became clear there is a wide disconnect between the state's definition of a strip search and what actually occurs during a strip search in prisons. For example, women who are menstruating are forced to remove their tampon or pad to show guards/police, which has been documented by activists and scholars (Davis, 2003; Hutchison, 2019; Kilroy, 2003; McCulloch & George, 2009; Ritchie, 2017; Roberts, 2020; Shakur, 1987). Additionally, women are forced to lift their breasts, spread their buttocks with their hands, squat, and cough, none of which are described in the legislation or policy.

These discrepancies in what legislation/policies state and what is practiced on the ground highlight the essentialness of speaking to the people who are on the receiving end of the policy. In doing so, a more nuanced and accurate understanding of what happens during a strip search, as well as women's experiences of it, can be elicited. Thus, the following section describes women's experiences of strip searching from their own positionalities, and places this in conversation with various literatures.

### Strip Searching is Sexual Violence

The findings of this research confirm what activists and scholars with lived experience of strip searching (e.g., Davis, 2003; Kilroy, 2003; Shakur, 1987) have been saying for decades – strip searching is sexual violence. My research builds on this and offers additional insights into the gendered, misogynoiristic, and colonial genocidal nature of strip searching.

Women I spoke with shared their experiences of being forced to remove their clothes; perform actions with intimate body parts such as their buttocks and breasts; and touch themselves in ways they did not want to, such as inserting a tampon without an applicator and spreading their buttocks apart with their hands while bending over. For example, during our first circle round in which women were asked to share anything they would like about strip searching, Beverly, a Métis mother and advocate, declared:

It's sexual assault. I don't care what anybody says. It's against what I want, it's my body, I'm naked. You are telling me to touch myself in ways I do not want to touch myself. And you are right in there looking? It's sexual assault.

This behaviour, if done in any other context outside of state power, would be considered sexual violence (Davis, 2003; George, 1993; Hutchison, 2020; Kilroy, 2003; McCulloch & George, 2009). Indeed, women in my research explicitly stated if anyone else forced them to strip naked and perform actions with intimate body parts it would be sexual violence. When I was speaking with Chantel, a mother and advocate who is in the process of determining her ancestry, about being forced to take her clothes off, she stated, “If somebody did that to me on the street, it's rape, it's sexual harassment.” Similarly, Dayna, who described herself as “a mix of races, including part Carib tribe, the Indigenous people of Trinidad” poignantly described strip searching in this way:

It's sexual abuse and it's mentally draining…I would say yes, it is definitely a form of abuse because you're not giving consent, you're being forced. Anybody on the street, if your partner or something, is telling you to spread your legs and everything you don't want to, and they're forcing that, that would be abuse and that would be something that you can charge them with.

Layering onto the descriptions above, in our one-on-one conversation, not only did Victoria, a white graduate student and advocate, emphatically state that strip searching is sexual assault, she also highlighted the additional harms of people being forced to remove their tampon during a strip search:

I definitely believe it's a form of sexual assault, 100%. And that's without the whole layer of if they've made somebody take out their tampon, or if you're wearing a pad, how devastating that would be. Or people's underwear, them inspecting them, just awful.

#### Being Strip Searched While Menstruating: “Oh, That's the Worst”

A common sentiment about being strip searched while menstruating was that it is “the worst.” Several women described it in this way, with Alia, a white mother and advocate, summarizing it aptly:

As for the whole period thing, I guess, what else do we have to really say besides, “Hey, people stripped us naked and watched us remove our tampons and cough blood all over the floor.” And what else really? How worse can it be?

Chantel shared how being strip searched while she was on her period was dehumanizing and degrading:

Well, yeah like, “Remove your tampon,” and then you have to put the new tampon in, in front of them. If you're in an institution that has tampons. And giving them your pad so they can check the sticky part of the pad to see if you have anything between your pad and your underwear. It's dehumanizing. It's degrading.

Like Chantel, Beverly recounted how she was not only forced to remove her tampon in front of guards, but she was also forced to insert a new one:

Yeah, it's degrading, right? If there's a worse word to describe it…But in federal when they're strip searching you on your moon time, you're going to the toilet and the prisons where I was at, we didn't have normal tampons because you get the OB ones without the applicators, whatever. But anyways, you're sitting on the toilet and you have a guard standing at the door, for me anyways, and watches you pull it out to make sure that you're actually pulling out a tampon. Then you got to stand up and you're not allowed to wipe or anything. And then they got to look at the tampon and make sure that it's a tampon. And then they flush the toilet and then you're left to put a new tampon in or a new pad while they're standing there with the door open because they want to be sure that nothing funny, no funny business happens. So many times.

Women with lived experience of strip searching (Davis, 2003; Kilroy, 2003; Shakur, 1987) along with other scholars (Hutchison, 2019; McCulloch & George, 2009; Ritchie, 2017; Roberts, 2020), have described situations whereby women have been made to remove their tampons and subsequently drip blood on the floor, as did women in this research. My research adds to the conversation by illuminating a nuanced component of strip searching while menstruating – the forced re-insertion of tampons without applicators while under surveillance of state agents.

The type of tampon some prisons give women after strip searching are ones without applicators, which require women to insert it using their finger. That women are being forced to insert their own finger into their vagina while agents of the state watch them constitutes state-inflicted sexual violence. Even if a tampon has an applicator, that women are forced to insert it into their vagina under surveillance of guards is a form of sexual violence perpetrated by the state. This practice is gendered in that only people who menstruate are forced to stick their fingers inside their vagina during a strip search. This is an additional layer of harm women, trans, and non-binary people experience during strip searches that cisgender men do not. Furthermore, the strip searching of Indigenous women during their moon time (menstruation) is not only gendered violence but an act of gendered *colonial* violence.

#### Strip Searching During Moon Time is Gendered Colonial Violence

Being strip searched during moon time was a significant source of conversation for most Indigenous women I spoke with. For example, Jessie, a Sioux mother and advocate, indicated that being strip searched during her moon was the most harmful aspect of strip searching for her, particularly when at an Indigenous healing lodge, and named it at the very beginning of her sharing (and even jumped ahead of the circle order to do so). She said:

The biggest … one of my things which got me in a lot of trouble, when I was at Okimaw Ohci Healing Lodge…it's like the best healing centre you can go to for federal women. Going in and being on your time [moon time], being on your time and having to get strip searched at the healing lodge. And these people are supposed to be teaching us stuff, teaching us our teachings. So I wouldn't, I didn't, and that would get me shit over, and over, and over.

Jessie's reflections bring to light the gendered colonial spiritual violence of strip searching Indigenous women during their moon time on several levels. Firstly, strip searching Indigenous women during their moon is a violent colonial act as this is a time of great spirituality and sacredness during which nobody should interfere (Anderson, 2016; MMIWG Inquiry, 2019). This spiritual violation replicates and reinforces the euro-western colonizers’ beliefs that:

Saw menstruation not a manifestation of female power, but as a manifestation of female sin, contamination, and inferiority. Missionaries did not understand menstruation as a sacred gift; rather, they taught women to see it from Western eyes— as a “curse.” (Anderson, 2016, p. 51)

These logics of severing connections to spirit and culture during moon time were a central focus in Indian Residential Schools when Indigenous girls entered puberty. In many Indigenous cultures, such as Anishinaabek and Cree, first menstruation is a sacred time that brings with it many powerful medicines with ceremonies and teachings accompanying this time (Anderson, 2016). However, when Indigenous girls were imprisoned in Residential Schools, these teachings were lost, and many girls did not know what was happening to them when they experienced their first moon, with some thinking they had cut themselves or were dying (Anderson, 2016; MMIWG Inquiry, 2019). Girls were also punished for not preventing their moon from getting on their sheets, despite not being given menstrual products. For example, girls were forced to raise their dresses and pull down their underwear to expose their genitals to everyone when they were found to have gotten their period on their bedsheets (Anderson, 2016).

Indigenous girls were taught their moon made them dirty, which is an anti-Indigenous misogynist trope deployed to justify and maintain settler theft and occupation of Indigenous lands. By rendering Indigenous women and girls dirty, colonizers dehumanize them and position them as objects in the way of unfettered settler access to land, and who must be displaced, dispossessed, and disappeared (Anderson, 2016; MMIWG, 2019). The strip searching of Indigenous women during their moon time is predicated on the same colonial logics. For example, Niya, a Saulteaux grandmother and social worker, reflected:

I do recall being strip searched when I was on my menstrual and having to go to the bathroom to go take out the tampon. It's disgusting. And it's so degrading and humiliating and the shame and you can't rage, right? You can't be angry about it because when you do speak… Like when I did speak out, it's like I'm doing something wrong. It's victimizing the victim. They toss your belongings aside like you're a piece of garbage and you're standing there feeling like a piece of garbage.

Furthermore, it is not only Indigenous women who are being violated when being strip searched during their sacred moon time. I received the teaching from Dr. Kathy Absolon, Anishinaabe Knowledge Keeper and Professor of Indigenous Social Work, that moon time is a spiritual time when women's grandmothers come to visit, which means strip searching Indigenous women during their moon time is also violence against their grandmother. This is a significant contribution to the knowledge about the gendered colonial harms of strip searching.

#### Colonial Harms of Strip Searching: “You Just Smell the Medicines Leaving”

The impacts and legacy of these assimilating settler colonial projects are far-reaching and extend generations beyond those directly affected. Indeed, during our circles, a few women spoke about their experiences with residential schools and how it connects to strip searching. A poignant example of how intergenerational trauma affects family members of residential school survivors, Sophia, an Oji-Cree grandmother, offered the following in response to the opening prompt to share anything she would like about strip searching:

It's so hard to start, like at first I didn't have a problem with strip searching and I attribute that to my conditioning from my mom and dad's conditioning, in residential school. You obeyed. My spirit rebelled, but I didn't…And so I got naked. And I was asked by this guard, there was only one guard, to kneel on the metal bench, facing the wall. I didn't have to bend over, lift my boobies or anything, lift my arms. She just had me kneel on the bench. And she left. When she came back, she told me to put my clothes back on. I couldn't understand that. Was that a form of punishment? And the more that I find out about residential school, I can see the similarity. Maybe I was too, like being Native, maybe I was too Nish [Anishinaabe] for them. “Okay, we need to work on this one a little bit more. Get some white in her or something”. But that's one thing they couldn't take away. I had just found out about my culture. I am Native, I am Indigenous, and I have something to be proud of.

While Sophia did not attend residential school herself, both of her parents did, and what she shared demonstrates how she was conditioned to obey those in positions of authority as a result of this experience. This may be particularly true when the authority figure is a representative of the white settler colonial state. Furthermore, Sophia's experience of having to kneel on a bench during a strip search follows the same colonial logics of Residential Schools whereby Indigenous children, likely including her parents, were forced to kneel on their beds and recite Christian/Catholic prayers. This is unsurprising given the entrenchment of Christianity in prisons in Canada, which can be understood as an extension of the racist policy guiding the residential school system to ‘kill the Indian in the child.’ The treatment of Sophia in this manner is a clear example of how this goal of the settler state continues to be enacted in prisons through the use of strip searching.

Beverly shared her experiences of being strip searched after attending Sun Dance and being forced to remove ceremonial clothing. Here is what she said:

One of the things about this is you have an Indigenous woman coming back from ceremony, myself included in the ceremony, you get pulled into the staff washroom. And it's not just a room or anything, it's the staff washroom. So again, it's a dirty area, gross. And you're standing in this big open area and you are told to take out your braid. So you take out the braid, you take out the feather, you shake your hair. So there goes that piece. You have to check your ears, you have to open your mouth. And then you're taking off your ceremonial clothes. So you have your ribbon skirt and instead of hanging it up nicely, they're taking it and they're shaking it and checking everything and they throw it off to the side onto the ground. For me, you just smell the medicines leaving. You just smell everything good that you felt about that day going with those guards as they're strip searching you.

The act of being forced to remove her braid, feather, and ribbon skirt, which guards threw on the dirty floor, rather than treating the skirt with respect, literally and figuratively strips Indigenous women of their identity as Indigenous women, and in Beverly's case, her Métis identity. Like the Indian Act (1876) which is a gendered colonial project to strip Indigenous women of their status and identities, the contemporary use of strip searching on Indigenous women in prison, which is enshrined in legislation (CCRA, 1992) and policy (CSC, 2017) developed and implemented by the state, is another such legal weapon used to attack Indigenous women.

While both Indigenous men and women are targets of colonial state violence, in order to dominate and eradicate Indigenous nations to gain access to their land, early colonizers recognized that the subjugation of Indigenous women was necessary (Deer, 2015). This logic persists today. Indeed, the MMIWG Inquiry (2019) declared that Indigenous women are the “heart of their Nations and communities” and their protection is crucial for the survival of Indigenous nations (p. 129). The mass incarceration of Indigenous women is not an accident; it is an intentional gendered colonial strategy to eliminate Indigenous peoples so the Canadian settler state can have access to their lands, resources, and waters. This research expands the scholarship on the colonial mechanisms through which Canada is committing genocide against Indigenous women (MMIWG Inquiry, 2019) by showing strip searching as one such gendered colonial tactic to dispossess and disconnect Indigenous women from their bodies and lands.

Similarly, misogynoiristic logics are embedded within the practice of strip searching as detailed next.

#### Black Women's Bodies as Dangerous: “Apparently, My Hair is Contraband”

Lorraine, a Black mother and advocate, recounted how her body, specifically her vagina, was treated by guards as though it were dangerous. She reflected:

One Sunday I had gotten back to the prison from an outing to the Catholic church. I remember getting strip searched. And after I took off all of my clothes and then you were supposed to bend over and touch your toes. So bending over and touching my toes. Of course when you go to the washroom, you're going to wipe your vagina. And so I had wiped and there was remnants of the toilet paper leftover outside my vagina. And the guard that was strip searching me said “Oh, is that cocaine?” And I'm like, “excuse me?” I have never been so livid.

Beginning with the transatlantic slave trade, Black women's bodies have been stigmatized and associated with being chattel, vessels to transport drugs, aggressive, threatening, criminal, impervious to pain, sexually available, unfeminine, and of superhuman strength to justify genocide and slavery through their stolen labour (Collins, 2000; Cooper, 2006; Davis, 2003; Hartman, 1997; Jones-Rogers, 2019; Maynard, 2017; Reece, 2010; Ritchie, 2017). Indeed, Lorraine's experience of being suspected of smuggling cocaine in her vagina when a guard saw pieces of toilet paper on her labia during a strip search suggests the misogynoiristic tropes of Black women as dangerous and drug smugglers structure guards’ assumptions during strip searching.

Living in a white supremacist, anti-Black country, Black women are at increased risk of state agents using physical and sexual force against them (Maynard, 2017; Ritchie, 2017). This is likely even more true for *imprisoned* Black women as they are assumed to be vessels to smuggle in drugs and other contraband, even if they are not imprisoned for drugs or have ever been caught smuggling them in. This was the case for Lorraine who was assumed to be smuggling in cocaine as the guard saw a white substance on the outside of her vagina while she was being strip searched. Rather than considering the possibility of it being something else (which it was), the misogynoiristic assumption was that Lorraine was concealing cocaine in her vagina. While Lorraine was able to extract herself from the situation without enduring further harm, the ever-present threat of violence for Black women at the hands of state agents was likely at the front of her mind.

Similarly, Kamille, a Black businessowner, described the misogynoiristic treatment she endured when she was first admitted to a federal prison directly from a provincial jail:

When I first got to [name of federal prison] I had in single braids, so similar to what I have in now. Of course, if you've seen any Black women with single braids, that hairstyle alone takes like four hours to do. The guard had me sit there and pull out every single braid in my hair…I had to cut it because the way it was, I couldn't just unravel it with my fingers. Every single braid… Because, apparently, my hair is contraband.

Black women's hair has been uniquely criminalized through white supremacy and as Kamille experienced, it plays out in specific ways during strip searches. Since the beginning of the transatlantic slave trade, Black women's hair has been used as a site through which to strip them of their identity, their culture, and connection to their homeland (Hartman, 1997; Powell, 2018). As the Legal Defense Fund (2022) in the United States explains:

Black hair is also an expression of identity and culture. It's a representation of history and carries deep emotional significance. Historically, Black hair has carried a profound symbolism. Cornrows, locs, twists, afros, bantu knots, and more all have historic connections to Black pride, culture, religion, and history. (Why is Black hair special, para 2)

Recognizing this, one of the first acts slave traders engaged in when stealing Black people from the shores of Africa was to shave their heads, which functioned to debase and dehumanize them and turn them into chattel (Powell, 2019). Furthermore, shaving enslaved people's heads was often used as a form of punishment. This logic of anti-Black dehumanization was experienced by Kamille. This was an attempt by prison authorities to denigrate Kamille and render her subordinate in the racialized prison hierarchy.

#### Role of Women Guards

The requirement for people to be strip searched by guards of the same sex is rooted in heteronormative values and beliefs (Standing Senate Committee on Human Rights, 2021) as it ignores the role power plays in sexual violence and strip searching by assuming women cannot commit sexual violence against other women. However, the findings from this search reveal women experience strip searching as sexual violence, even when strip searched by women guards, regardless of the race of the guard.

Furthermore, it does not take into consideration the historical harm white women have caused Black and Indigenous women and girls such as during slavery and residential schools, which is important given many prison guards in Canada are white. As Jones-Rogers (2019) has meticulously detailed, white women inflicted horrendous violence on Black women and girls during slavery. Not only did white women wield their power and dominance in their household by abusing enslaved people, they also actively participated in the purchase and sale of Black people, which is a fact that has been largely invisibilized in dominant narratives of slavery. For example, when a Black enslaved woman “continually failed to become pregnant, her mistress had her stripped naked and whipped her severely” (Jones-Rogers, 2019, p. 23).

Similarly, women actively participated (and still do) in the attempted assimilation, dispossession, and genocide of Indigenous nations in service to settler colonialism. White women missionaries imposed Christian hierarchies or patriarchy, social workers tore Indigenous children from their families during the residential school era and currently do through the child welfare system and white nuns also violently abused Indigenous children, including sexual violence.

Not only are all guards “agents of domination” (Davis, 1981) and “enforcers of white supremacy” (Ritchie, 2017) under the guise of public safety, white women guards strip searching Indigenous and Black women is a particular demonstration of settler colonial and misogynoiristic logics enacted in residential schools and during slavery. The violence they are enacting on Indigenous and Black women imparts centuries old logics of racial superiority and domination and functions to maintain the patriarchal and white supremacist hierarchy our society is built upon. Women guards, regardless of their race, maintain their position in the hierarchy by subjugating imprisoned women to ensure they remain beneath them. However, this actually functions to strengthen white patriarchal domination over all women in that rather than fighting against the logics of white supremacist patriarchy (hooks, 1984) to free all women from its clutches, women guards are enacting these logics thereby strengthening its grip.

## Discussion of Key Implications and Contributions

One of the most significant contributions of this research is that it conceptualizes misogynoiristic and colonial genocidal violence as embedded within strip searching. Rather than being understood exclusively through a gendered analysis, this research highlights the ways in which strip searching is rooted in legacies of settler colonialism, white supremacy, and anti-Black racism and the ways in which they interlock with misogyny.

### Practice

A main contribution of this research is that it corroborates what women with lived experience of strip searching have been saying for decades – strip searching is sexual violence (Davis, 2003; Kilroy, 2003). This has important implications for social work given the profession's mandate to strive towards social justice and human rights (International Federation of Social Work, 2019). Sexual assault support centres, domestic violence shelters, halfway houses, and any agency supporting women who have been imprisoned, should include strip searching in their conceptualization of sexual violence and provide the appropriate supports therein.

However, Black and Indigenous women are not often centered in spaces that support victims/survivors of sexual violence. Mainstream nonprofits are often built upon white, middle-class norms, which result in a lack of support for anyone outside of these bounds. This is particularity true with respect to Black and Indigenous women who have been incarcerated due to white supremacy, misogynoir, and anti-Indigenous racism. Mainstream nonprofits can use this research to unpack the ways in which their presumptions structure who qualifies as a victim/survivor of sexual violence, and how this influences the services and supports they offer.

Social workers who provide counselling in carceral settings would better support people by acknowledging the state is enacting sexual violence against them in the form of strip searches. As Pollack (2007) highlights, validating women's experiences of sexual harms by the state is one way to politicize one's practice and support women to make decisions in the context of incredible power imbalances. Social workers can support women by meeting their direct needs after being strip searched while simultaneously advocating for an abolishment of strip searching. Indeed, this is at the core of abolition feminist praxis in that work must be done at both micro and macro levels by attending to women's immediate needs while also pushing for structural change (Davis et al., 2022; Richie & Martensen, 2020).

### Policy and Legislation

Legislators and prison authorities can use this research as impetus to change the legislation and policies relating to strip searching as there is no law in Canada indicating strip searching *must* be conducted. Individual prison administrators can choose to implement a search plan for their prison that does not incorporate strip searching as, by law, they have the discretion to do so. Furthermore, legislators can decide to abolish the use of strip searching. Women's stories were replete with examples of how the practice of strip searching is gendered and rooted in logics of misogynoir and colonial genocide, and function to strengthen and maintain the white supremacist settler colonial state. As abolition movement leaders Kaba and Ritchie (2022) state, in order to eradicate sexual violence, we need to eliminate sexual violence by the state, which includes the use of strip searching.

## Limitations

The major limitation of this study is that it did not gather experiences from trans, non-binary, and Two-Spirit people who have been previously imprisoned. This was not an intentional research design; however, I could have done more to ensure gender diverse and Two-Spirit people felt safe enough to participate. Going forward, all my research into conditions of confinement will be inclusive of everyone who is incarcerated in prisons designated for women.

Although I believe holding the circles and conversations virtually allowed for a more expansive scope that did not focus exclusively on Ontario, I wonder how being in circle in person, sharing food, and having informal conversations before and after the circle would have changed the story gathering.

## Conclusion

This study confirms what women with lived experience have been saying for decades (Davis, 2003; Kilroy, 2003; Shakur, 1987) – strip searching is sexual violence. That this violence is legislated and enacted by the state and its agents renders strip searching state-inflicted sexual violence. Women shared their experiences of being forced to remove their clothes and perform actions with intimate parts of their bodies, which included touching themselves in ways they did not want to, in front of women agents of the state. The harms of this sexual violence were amplified when women were strip searched while they were menstruating as they were forced to remove their tampons or pads and show guards, which is consistent with what activists and scholars have been arguing (Davis, 2003; McCulloch & George, 2009).

In addition to the gendered harms of strip searching, this study elucidates how logics of settler colonialism and misogynoir are embedded within the practice of strip searching. Indigenous women shared their experiences of being strip searched after attending ceremony and during their moon time, which is a spiritual violation. It also drew connections between the settler colonial sexual violence perpetrated at Indian Residential schools and the use of strip searching. Similarly, Black women shared their experiences of being assumed to be smuggling drugs into the prison through their vagina and their hair, which is consistent with misogynoiristic tropes that Black women are transporters of drugs (Collins, 2000; Maynard, 2017; Richie, 2012; Ritchie, 2017). It also drew parallels between the institutionalized sexual violence during slavery and the use of strip searching.

Given the demonstrated harms of strip searching, and for social work to live up to its mandate to strive for social justice and human rights (IFSW, 2019), social workers and the discipline at large have an obligation to advocate for the abolishment of strip searching in prisons and all carceral settings in which social work is implicated. Indeed, every woman in this study called for the abolishment of this violent practice.

## Notes

1. While the Corrections and Conditional Release Act (1992) describes contraband as an intoxicant, weapon, money, or any item that could jeopardize the security of the institution, in practice it is used as an all-encompassing descriptor of any item found, including those outside the parameters of the legislated definition. This is an intentional strategy to inflate the data to give the impression more “dangerous” items were found than is the case, thus justifying the use of strip searching and other carceral practices.

2. Following the lead of CAEFS, I use the term “prisons designated for women” because so-called “women's prisons” incarcerate women (inclusive of trans women), trans men, non-binary, and Two-Spirit people who do not fit into colonial notions of gender binaries (CAEFS, 2021).

3. This research received University Research Ethics Board approval.

4. Since then, one participant has indicated she is currently trying to learn more about which Nation she comes from and thus does not currently identify as Indigenous. Until she can confirm this, she is identifying as “a human with a purpose and a will to live.”

5. This participant described herself in this way, “I am a mix of races, I am Trinidadian and am part Carib tribe, the Indigenous people of Trinidad.” She participated in a circle with Black women, which included another woman from Trinidad.

6. Dr. Absolon is the founder of the Centre for Indigegogy, which offers decolonial workshops and training. I have completed the Advanced Decolonizing Education Certificate (DEC), Indigenous Research Series, Intro to Circle Work, and Facilitating Decolonial Circles. I am also a Research Associate with the Centre and co-facilitate Module 1 of the DEC with Dr. Absolon called Introduction to Indigenous perspectives and knowledge: Cultural history and colonial history. It is through these trainings, along with Absolon (2011, 2022), that I have learned how to facilitate talking circles following Anishinaabe protocols.
